# Association Between Electronic Patient Portal Enrollment and HIV Care Outcomes Among People Living with HIV at an Urban Academic Medical Center

**DOI:** 10.1007/s10461-025-04869-7

**Published:** 2025-08-29

**Authors:** Jessica P. Ridgway, Daniela Zimmer, Raj Shetty, Neda Laiteerapong

**Affiliations:** 1 Department of Medicine, University of Chicago, Chicago, IL, USA; 2 Pritzker School of Medicine, University of Chicago, Chicago, IL, USA

**Keywords:** Electronic patient portal, Retention in care, HIV engagement in care

## Abstract

Electronic patient portals are associated with improved health outcomes in primary care settings. We assessed the relationship between patient portal enrollment and retention in care and HIV viral suppression in an urban HIV care clinic. In multivariable models, people with HIV with an active portal account had higher odds of HIV viral suppression (Odds Ratio [OR]: 2.02, 95% CI [1.3–3.7]) and higher odds of retention in care (OR: 2.2 95% CI[1.4–3.4]) than those without an active portal account. Our findings suggest that portal enrollment is associated with improved HIV care outcomes among people with HIV.

## Introduction

Patient portals are secure websites, or web-based applications, that give patients access to their health information from anywhere with a web connection [1]. Portals allow patients to view test results, securely message their providers, request prescription refills, and schedule medical appointments. Portals have become increasingly available with 84% of individuals in the U.S. reporting being offered the ability to access their online medical record or patient portal in 2022 [2].

Portal use has been associated with improved patient engagement in care, better patient-provider communication, and improved health outcomes in primary care settings [1]. Among people with HIV (PWH) who have active patient portal accounts, use of certain portal features has been associated with engagement in HIV care. For example, viewing scheduled appointments in the portal is associated with appointment attendance [3], and use of the portal to request antiretroviral therapy (ART) refills is associated with ART adherence [4]. However, to date, only one study has examined whether portal use was associated with HIV-related outcomes, like retention in care and viral suppression. Thus, we sought to assess the relationship between patient portal enrollment and these HIV-related outcomes.

## Methods

This project took place in an adult Ryan White HIV care clinic in Chicago. IL. Our institution utilizes Epic as an electronic medical record, and MyChart as the electronic patient portal. MyChart enrollment is routinely offered to all new patients and any patients without an active MyChart account during check-in at outpatient clinic appointments. Patients who are interested in enrolling in MyChart are provided with information regarding how to set up a MyChart account.

All patients 18 years of age or older with a diagnosis of HIV who attended an appointment in the HIV care clinic between April 25, 2022 and April 24, 2024 were included. We collected electronic health record data for these individuals, including demographic characteristics, HIV viral load, CD4 count, dates of HIV care encounters, and MyChart enrollment status (active vs. inactive). This project received a formal Determination of Quality Improvement status according to institutional policy. As such, this initiative was deemed not human subjects research and was therefore not reviewed by the Institutional Review Board.

Patients were determined to have HIV viral suppression if their most recent HIV viral load was less than 200 copies/mL. Retention in care was defined using the six-month gap definition [5], i.e., PWH who attended an HIV care appointment in the prior 6 months were considered retained in care. We compared characteristics of PWH with an active MyChart account vs. PWH without an active MyChart account using Student’s t-test for continuous variables and Chi-squared test for dichotomous variables. We created multivariable logistic regression models to assess if MyChart status was associated with three different HIV care outcomes: HIV viral suppression, retention in care, and CD4 count > 200 cells/mm^3^, after controlling for age, race, and sex. P-values less than 0.05 were considered significant. Analyses were performed using R version 4.4.0.

## Results

785 PWH were included in the analysis, of whom 670 (85.4%) had an active MyChart account. The mean age was 47 years (SD = 14.9), 70.3% (552/785) were male, and 82.7% (649/785) were Black or African American. Table 1 shows results of bivariate associations between patient characteristics and patient portal account status. While sex was not associated with portal account status, those without active MyChart accounts were more likely to be older and Black race than those with active MyChart accounts.

In the multivariable model for the outcome of HIV viral suppression, PWH with an active MyChart account had higher odds of HIV viral suppression (Odds Ratio [OR]: 2.02, 95% CI [1.3–3.7]) compared to those without an active account. In the multivariable model for the outcome of retention in care, PWH with an active MyChart account had higher odds of having had an HIV care appointment in the prior 6 months compared to those without an active MyChart account (OR: 2.2 95% CI [1.4–3.4]). Finally, in the multivariable model for the outcome of CD4 count, PWH with an active MyChart account had three times the odds of having a CD4 count greater than 200 cells/mm^3^ compared to those without an active MyChart account (OR: 3.3, 95% CI [1.8 to 6.0]).

## Discussion

Among people with HIV, we found that an active patient portal account was associated with positive health outcomes, including HIV viral suppression and retention in care. Not only are viral suppression and retention in care associated with improved health outcomes for PWH such as decreased risk of mortality, but PWH who are virally suppressed and retained in care are also much less likely to transmit HIV to others [6]. As our findings were observational, we were only able to investigate the association between portal enrollment and HIV care outcomes, and not causation. It may be that people who are already virally suppressed and engaged in their healthcare are more likely to utilize the portal, or that use of the portal itself promoted improved health outcomes.

Other observational studies have found that portal use in the general population is associated with a variety of positive health outcomes, including preventative health behavior such as influenza vaccination [7], and diabetes control [8]. Among PWH, Schember et al. found that having a portal account was associated with retention in care and viral suppression in an HIV care clinic in Tennessee from 2011 to 2016 [9]. Sukija-Cohen et al. examined the relationship between logging into a patient portal that allowed patients to review their upcoming appointment and laboratory results, and found that PWH who logged into the portal prior to their scheduled appointment were 7.4 times more likely to complete their first appointment than patients who did not log in [3]. While our project did not assess granular login activity, we did find that patients with portal access were more likely to be retained in care.

The patient portal can be utilized for variety of functions which could improve patients’ health. Beyond viewing appointments, portals can also be used to request prescription refills, securely message providers, and view laboratory test results. Midboe et al. examined the relationship between patient portal use and ART adherence and viral load suppression [4]. They found that use of the portal for prescription refills, secure messaging, or viewing appointments was positively associated with ART adherence [4]. In their study, viewing laboratory results was positively associated with viral suppression, but viewing appointments was not [4]. The portal can also be utilized to collect patient-reported outcomes such as anxiety and depression or social needs [10], and more studies needed to assess how portal-based collection of patient-reported outcomes impacts HIV care outcomes.

Prior studies have found conflicting results regarding the association between age and portal use, with some showing higher portal use among older individuals and others showing lower use among older individuals [1, 2]. We found that older people with HIV were less likely to have an active portal account than younger people. Similar to prior literature [2, 9], we also found that Black individuals were less likely to have an active portal account than white individuals. Hall et al. implemented an intervention to increase portal enrollment among Black PWH who were previously unenrolled in the patient portal [11]. 62% of their study population newly enrolled in the portal, but some portal non-users expressed general technological mistrust and wariness of using the internet to access personal healthcare information [11]. More research is needed to understand how to close the digital divide, and ensure equal access to portals for all users.

Our analysis has several limitations. We did not examine actual logins to the portal or use of specific portal functions, but rather whether individuals had an active account. We were only able to measure associations between portal activation and care outcomes, and not causation. It is possible that other confounders not measured may account for differences between portal users and non-users, such as social drivers of health.

In conclusion, our findings suggest that patient portal account use is associated with improved HIV care outcomes. More research is needed to understand ways to increase portal account access and to identify which specific functions of the portal are most important for HIV care.

## Figures and Tables

**Table 1:** Characteristics of patients with HIV with and without active portal accounts

Patient characteristics	Active portal account (*N* = 670, 85.4%) *N* (column %)	Inactive portal account (*N* = 115, 14.6%) *N* (column %)	Statistical test and value	*P* value
Sex				0.11
Male	464 (69.2%)	88 (76.5%)	Chi-squared = 2.48	
Female	206 (30.7%)	27 (23.4%)		
Age (years, mean (SD))	45.5 (14.5)	55.8 (14.7)	Student’s t-test = 7.02	< 0.01
Race			Chi-squared=9.85	0.02
African American/Black	543 (81.0%)	106 (92.1%)		
White	90 (13.4%)	4 (3.5%)		
Other/unknown	37 (5.5%)	5 (4.3%)		
HIV viral load			Chi-squared=7.88	< 0.01
<200 copies/mL	472 (70.4%)	66 (57.4%)		
≥ 200 copies/mL	183 (27.3%)	46 (40.0%)		
No viral load information	15 (2.2%)	3 (2.6%)		
Retention in HIV care			Chi-squared=7.55	< 0.01
Encounter in prior 6 months	378 (56.4%)	49 (42.6%)		
No encounter in prior 6 months	292 (43.6%)	66 (57.4%)		
CD4 count			Chi-squared=17.13	< 0.01
≥ 200 cells/mm^3^	595 (88.8%)	90 (78.3%)		
<200 cells/mm^3^	54 (8.1%)	24 (20.9%)		
No CD4 count information	21 (3.1%)	1 (0.9%)		

Abbreviations: HIV, human immunodeficiency virus; SD, standard deviation

## Data Availability

Deidentified data are available upon reasonable request to the authors.
